# Deep eutectic solvents for antiepileptic drug phenytoin solubilization: thermodynamic study

**DOI:** 10.1038/s41598-021-03212-z

**Published:** 2021-12-16

**Authors:** Hemayat Shekaari, Mohammed Taghi Zafarani-Moattar, Masumeh Mokhtarpour, Saeid Faraji

**Affiliations:** Department of Physical Chemistry, University of Tabriz, Tabriz, Iran

**Keywords:** Pharmaceutics, Thermodynamics

## Abstract

Thermodynamic investigations provide information about the solute-solvent interactions in the selection of the proper solvent for different fields of pharmaceutical sciences. Especially, the study of antiepileptic drugs in solutions (ethanol/co-solvent) has been a subject of interest owing to their effect in the systems using interaction with a number of important biological membranes. This work focuses on the measurement of density and speed of sound of the phenytoin (PTH) in ethanol/deep eutectic solvents (choline chloride:ethylene glycol, and choline chloride:glycerol) solutions as the innovative class of green solvents at temperature range (288.15 to 318.15) K. It was determined Hansen solubility parameters for assessment of PTH interactions in the solvent media. Some thermophysical parameters including apparent molar volumes *Vϕ*, apparent molar isobaric expansion $E_\varphi^0$, and Hepler’s constant, apparent molar isentropic compressibility *κ*_*φ*_ were obtained and calculated using these data. To correlate  the *Vϕ* and *κ*_*φ*_ values, the Redlich-Meyer equation was used to calculate the number of quantities containing standard partial molar volume and partial molar isentropic compressibility. Finally, $\Delta \delta$ values showed a strong interaction between PTH and solvent (ethanol/DES (ChCl:EG)). The thermodynamic analysis of the studied system also plays a crucial role in the pharmaceutical industry.

## Introduction

**Figure 1:**
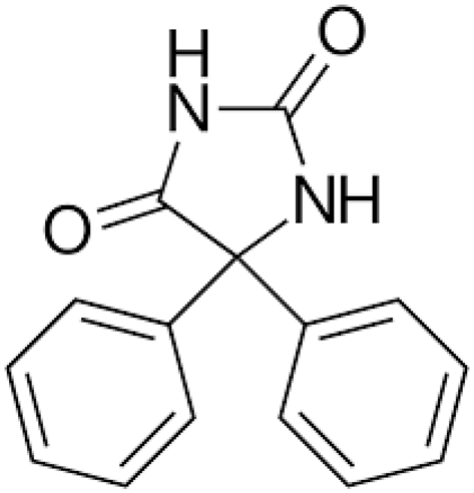
Molecular structure of Phenytoin (PTH).

Extraction and recrystallization of pharmaceutical compounds are, by far, the most important step in the drug manufacturing processes. Poor solubility is a chief limitation to oral delivery of numerous emerging drugs and bioavailability is significantly affected by the drug solubility^1,2^. Phenytoin (PHT, Fig. 1) is an anti-epileptic drug, which is applied in the therapeutics. Phenytoin (PHT) is introduced as an anti-seizure drug as well as is proper for the snub of focal seizures and, tonic–clonic seizures but not absence seizures. It can also be utilized for some neuropathic pain or heart arrhythmias. It can be used mouth or intravenously^3^. The intravenous form generally begins within 30 min and is operational for 24 h. Blood levels can be measured to distinguish the appropriate dose^4^. This drug is categorized as a hydantoin derivative and despite its narrow therapeutic index, it is one of the most commonly used anticonvulsants. In addition, its applications are numerous such as an effective anti-epileptic, bipolar disorder, retina protection, and wound healing^5^. Low solubility of PHT has always presented major obstacle towards the development of extraction, re-crystallization and so drug delivery systems and the low solubility of PHT indicated the need of use the other solvents in these steps^6,7^.

The most common co-solvents for pharmaceutical compounds are organic solvents. However, the organic solvents applied in various sciences are usually flammable, toxic and volatile^8,9^. In contrast to conventional organic solvents, ionic liquids (ILs) and deep eutectic solvents (DESs) are considered environmentally benign “green and sustainable” solvents^10^. However, DESs exhibit similar physical and chemical properties of ILs and mostly DESs, especially the DESs used in this work are inexpensive to prepare, much less toxic, and are biocompatible and biodegradable. These green solvents were introduced and defined in 2003 and have many attractive potential applications in several fields^11,12^.

Solubility of several drugs in the presence of various DESs has been reported in our previous works, and the results show a significant increase in the solubility of drugs.

On the other hand, understanding the interactions of drugs in the solvent mixtures has been a topic of research to extract them from the basic media. Physicochemical and thermodynamic studies also attract researchers owing to the significant performance of drugs. The nature and the extent of the patterns of molecular interactions that exist in mixtures can be studied via physicochemical and thermodynamic investigations^13,14^. Thus, this research was aimed to represent the continuation of a systematic investigation of the volumetric properties of PHT in solvent mixtures at various temperatures *T* = (288.15, 298.15, 308.15 and 318.15) K. The derived thermophysical parameters including the apparent molar volume, ${V_\phi }$, standard partial molar volume,$V_\phi^0$, apparent molar isentropic compressibility, *κ*_*φ*_, and infinite dilution apparent molar isentropic compressibility,$\kappa_\phi^0$ values. Finally, Hansen solubility parameters for assessment of PTH interactions were used in the solvent media. These parameters can help to predict the solvent performance during the manufacturing processes and will be useful in explanation of solvent behavior in many other fields. The obtained parameters were used to survey the impact of the DES on the solute–solvent interactions in the systems of PHT.

## Results and discussion

### Density and speed of sound results

**Figure 2:**
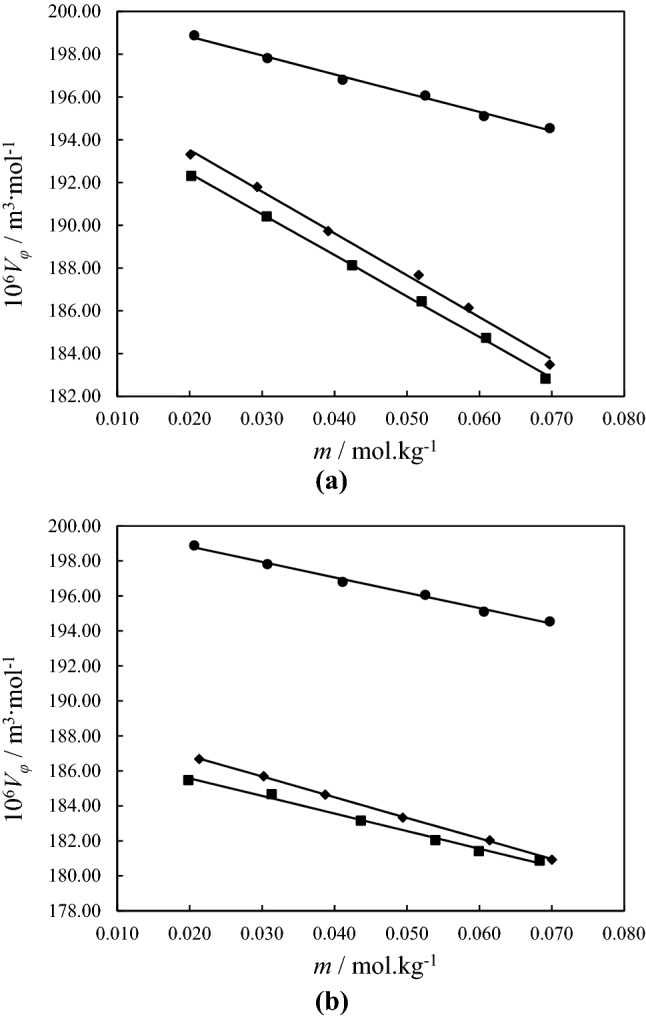
Apparent molar volumes,${V_\phi }$, of PTH in binary PTH/ethanol solutions and ternary PTH/DESs/ethanol solutions at *T* = 298.15 K; (a), ternary PTH/DESs/ethanol solutions with DESs molality = 0.5 mol/kg; (b), ternary PTH/DESs/ethanol solutions with DESs molality = 1.5 mol/kg; (filled black circle), binary PTH/ethanol solution; (filled black square), ternary PTH/ChCl:EG/ethanol; (filled black diamond), ternary PTH/ChCl:Gly/ethanol

The apparent molar volumes *Vϕ* of binary PTH/ethanol and ternary PTH/ethanol/DESs (ChCl:Gly, and ChCl:EG) in diverse DES molalities (0.5, 1, and 1.5 mol kg^-1^) were calculated using the measurements of density *d* data. In the studied systems, the PTH is defined as a solute, and DESs are introduced as co-solvent. From the data in Table 1, it can be seen that the densities decrease with increasing temperature. The Eq. (1) was used to calculate the apparent molar volumes *Vϕ*:

1 $${V_\varphi } = \frac{M}{d} - \left[ {\frac{{(d - {d_0})}}{{md{d_0}}}} \right]$$

where *M* (kg mol^-1^), and *m* (mol kg^-1^) are the molar mass and the molality of the PTH. The *d*_0_ (kg m^-3^) and *d* (kg m^-3^) are also density of solvent (ethanol and DESs + ethanol) and density of the solutions. The values of ${V_\varphi }$ for the mentioned systems at all worked temperatures are given in Table 1. For the binary PTH + ethanol and ternary PTH/ethanol/DESs solutions, the ${V_\varphi }$ values have a downward trend at all temperatures. Figure 2 indicate the ${V_\varphi }$ values for binary PTH + ethanol and ternary PTH/ethanol/DESs (with molalities 0.5 and 1.5 mol kg^-1^) solutions at* T* = 298.15 K. The positive values of ${V_\varphi }$ decreased with rising of the PTH molalities. The reduction in the values of ${V_\varphi }$ with increasing temperature causes more attraction for DESs, which is evidence of strong interactions between PTH and solvent. According to the calculated results, it is clear that the values of ${V_\varphi }$ also decreased with increasing DES amount. This behavior may be due to the attenuation of the interactions between PTH and the ethanol molecule that occur by increasing the concentrations of DESs. The intermolecular forces between PTH and ethanol are reinforced due to functional groups and various ionic groups in DESs.

**Table 1:** The density (*d*) data and apparent molar volume (${V_\varphi }$) values for PTH molalities *m*_PTH_ (mole of PTH per 1 kg of ethanol for binary system and mole of PTH per 1 kg of DESs/ethanol solutions for ternary system) in binary PTH/ethanol and ternary PTH/DESs (ChCl:Gly and ChCl:EG)/ethanol solutions at *T* = (288.15 to 318.15) K and ambient pressure (*P* = 871 hPa).

*m /* mol kg^-1^	10^–3^ *d* / kg m^-3^				10^6^${V_\varphi }$/ m^3^ mol^-1^			
*T /* K	288.15	298.15	308.15	318.15	288.15	298.15	308.15	318.15
**PTH in Ethanol**								
0.0206	0.795161	0.786702	0.777995	0.769197	200.21	198.89	197.64	196.37
0.0307	0.795919	0.787475	0.778778	0.769989	199.24	197.82	196.70	195.59
0.0411	0.796717	0.788287	0.779602	0.770821	198.23	196.81	195.67	194.69
0.0525	0.797591	0.789176	0.780517	0.771746	197.48	196.07	194.52	193.56
0.0606	0.798223	0.789823	0.781167	0.772410	196.63	195.11	193.76	192.62
0.0697	0.798931	0.790544	0.781894	0.773154	196.09	194.55	193.33	192.01
**PTH in ternary ethanol solution of ChCl:Gly (0.5 mol kg**^**-1**^**)**								
0.0000								
0.0201	0.810656	0.802444	0.793945	0.785270	195.39	193.32	191.31	189.82
0.0293	0.811379	0.803183	0.794700	0.786041	193.85	191.80	189.75	188.02
0.0391	0.812170	0.804000	0.795532	0.786895	192.15	189.73	187.72	185.63
0.0516	0.813203	0.805059	0.796636	0.788012	190.16	187.68	184.89	182.99
0.0585	0.813801	0.805670	0.797236	0.788647	188.63	186.15	184.03	181.42
0.0697	0.814791	0.806701	0.798281	0.789699	186.43	183.49	181.44	179.12
**PTH in ternary ethanol solution of ChCl:Gly (1 mol kg**^**-1**^**)**								
0.0000								
0.0202	0.824831	0.816802	0.808351	0.799892	192.34	190.05	188.65	187.00
0.0306	0.825657	0.817652	0.809218	0.800772	190.89	188.43	186.86	185.30
0.0423	0.826626	0.818637	0.810225	0.801790	188.66	186.47	184.69	183.28
0.0519	0.827441	0.819465	0.811072	0.802655	186.91	184.84	182.92	181.29
0.0596	0.828109	0.820158	0.811775	0.803356	185.59	183.21	181.32	180.00
0.0697	0.829002	0.821081	0.812688	0.804292	183.86	181.23	179.89	178.28
**PTH in ternary ethanol solution of ChCl:Gly (1.5 mol kg**^**-1**^**)**								
0.0000								
0.0213	0.838607	0.830687	0.822476	0.814247	188.23	186.68	184.50	183.05
0.0302	0.839322	0.831420	0.823224	0.815011	187.47	185.70	183.60	181.94
0.0387	0.840032	0.832140	0.823956	0.815749	186.27	184.65	182.68	181.26
0.0494	0.840936	0.833053	0.824888	0.816713	184.76	183.34	181.35	179.35
0.0614	0.841944	0.834084	0.825932	0.817771	183.62	182.04	180.21	178.34
0.0700	0.842721	0.834849	0.826702	0.818554	182.02	180.93	179.27	177.40
**PTH in ternary ethanol solution of ChCl:EG (0.5 mol kg**^**-1**^**)**								
0.0000								
0.0202	0.806469	0.797874	0.789627	0.780944	194.09	192.31	190.14	188.51
0.0306	0.807303	0.798724	0.790495	0.781826	192.19	190.42	188.23	186.59
0.0424	0.808281	0.799718	0.791511	0.782850	189.84	188.13	185.85	184.47
0.0520	0.809094	0.800553	0.792364	0.783728	188.42	186.45	184.12	182.27
0.0609	0.809858	0.801335	0.793149	0.784543	186.82	184.74	182.71	180.37
0.0691	0.810615	0.802094	0.793906	0.785305	184.68	182.83	181.12	178.95
**PTH in ternary ethanol solution of ChCl:EG (1 mol kg**^**-1**^**)**								
0.0000								
0.0202	0.816446	0.808535	0.800168	0.791795	190.60	188.49	186.83	185.05
0.0306	0.817297	0.809394	0.801044	0.792680	188.75	187.15	185.38	183.91
0.0423	0.818276	0.810391	0.802044	0.793700	186.93	185.26	184.00	182.31
0.0519	0.819095	0.811217	0.802890	0.794563	185.48	184.00	182.46	180.62
0.0596	0.819768	0.811901	0.803584	0.795257	184.28	182.76	181.20	179.64
0.0697	0.820683	0.812810	0.804493	0.796180	182.32	181.20	179.92	178.31
**PTH in ternary ethanol solution of ChCl:EG (1.5 mol kg**^**-1**^**)**								
0.0000								
0.0198	0.825866	0.818110	0.809971	0.801254	187.79	185.48	183.62	181.83
0.0313	0.826822	0.819067	0.810956	0.802249	185.98	184.68	182.34	180.91
0.0436	0.827856	0.820121	0.812015	0.803330	184.64	183.17	181.46	179.78
0.0539	0.828730	0.821015	0.812920	0.804255	183.70	182.05	180.44	178.58
0.0599	0.829255	0.821540	0.813449	0.804792	182.86	181.42	179.91	178.07
0.0683	0.829980	0.822265	0.814194	0.805547	182.09	180.88	179.17	177.37

Standard uncertainties (*u*) for each variable are *u* (*T)* = 0.001 K; *u* (*m*) = 0.0005 mol kg^-1^; *u* (*p*) = 10 hPa,

*u* (*ρ)* = 0.015 kg m^-3^.

**Figure 3:**
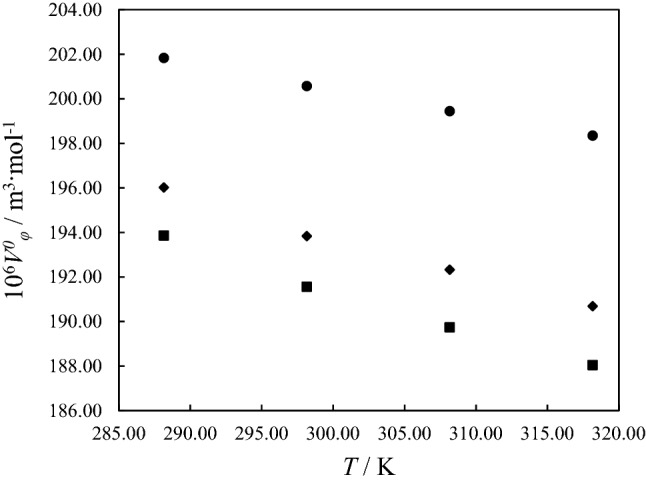
The comparison of the standard partial molar volumes,$V_\varphi^0$, of PTH in binary PTH/ethanol solutions and ternary PTH/DESs/ethanol solutions with DESs molality = 1 mol/kg at different temperatures: (filled black circle), binary PTH/ethanol solution; (filled black square), ternary PTH/ChCl:EG/ethanol; (filled black diamond), ternary PTH/ChCl:Gly/ethanol.

The following relation, known as the Redlich-Meyer equation, is used to determine the standard partial molar volume $V_\varphi^0$ for PTH^15^:

2 $${V_\varphi } = V_\varphi^0 + {B_v}m$$

where *B*_*v*_ is the empirical parameter of the equation. The least-squares analysis was used to obtain the $V_\varphi^0$ and *B*_*v*_ parameters, which were presented in Table 2. The obtained values of $V_\varphi^0$ represent the solute–solvent interactions. In Fig. 3, variations of $V_\varphi^0$ are demonstrated for each system at DESs molality *m* = 1 mol kg^-1^ versus the worked temperature. The obtained parameters show that the $V_\varphi^0$ values are similar to the ${V_\varphi }$ values decreasing with increasing temperature and decreasing with increasing DES molalities.

**Table 2:** The parameters, $V_\varphi^0$, *B*_*v*_, *∆*_*tr*_*V*^*0*^_*φ*_ along standard deviations $\sigma ({V_\varphi })$ for the binary PTH/ethanol and ternary PTH/DESs/ethanol solutions at *T* = (288.15 to 318.15) K and at ambient pressure (*P* = 871 hPa).

*T* / K	10^6^ *V*^*0*^_*φ*_ / m^3^ mol^-1^	10^6^ *B*_*v*_ / m^3^ kg mol^-2^	10^6^ *∆*_*tr*_*V*^*0*^_*φ*_ / m^3^ mol^-1^	*σ* (*V*_*φ*_)
**PTH in ethanol**				
288.15	201.84	− 84.14	–	0.13
298.15	200.58	− 88.03	–	0.15
308.15	199.45	− 91.11	–	0.17
318.15	198.35	− 91.79	–	0.12
**PTH in ternary ethanol solution of ChCl:Gly (0.5 mol kg**^**-1**^**)**				
288.15	199.11	− 178.97	− 2.73	0.21
298.15	197.44	− 195.52	− 3.14	0.30
308.15	195.46	− 199.76	− 3.99	0.25
318.15	194.26	− 218.22	− 4.09	0.13
**PTH in ternary ethanol solution of ChCl:Gly (1 mol kg**^**-1**^**)**				
288.15	196.02	− 174.54	− 5.82	0.13
298.15	193.84	− 177.88	− 6.74	0.20
308.15	192.33	− 180.89	− 7.12	0.14
318.15	190.69	− 178.60	− 7.66	0.11
**PTH in ternary ethanol solution of ChCl:Gly (1.5 mol kg**^**-1**^**)**				
288.15	191.11	− 126.55	− 10.73	0.23
298.15	189.21	− 117.86	− 11.37	0.05
308.15	186.82	− 108.20	− 12.63	0.07
318.15	185.54	− 117.68	− 12.81	0.24
**PTH in ternary ethanol solution of ChCl:EG (0.5 mol kg**^**-1**^**)**				
288.15	197.91	− 186.91	− 3.93	0.26
298.15	196.25	− 191.37	− 4.33	0.15
308.15	193.80	− 184.08	− 5.65	0.12
318.15	192.65	− 198.87	− 5.70	0.16
**PTH in ternary ethanol solution of ChCl:EG (1 mol kg**^**-1**^**)**				
288.15	193.87	− 163.62	− 7.97	0.13
298.15	191.57	− 147.81	− 9.01	0.10
308.15	189.75	− 141.07	− 9.70	0.14
318.15	188.05	− 140.08	− 10.3	0.17
**PTH in ternary ethanol solution of ChCl:EG (1.5 mol kg**^**-1**^**)**				
288.15	189.81	− 114.9	− 12.03	0.21
298.15	187.58	− 100.29	− 13.00	0.18
308.15	185.32	− 90.18	− 14.13	0.10
318.15	183.78	− 94.31	− 14.57	0.10

The partial molar transfer ${\Delta_{tr}}V_\phi^0$ is another essential quantity to express useful information about interactions. The ${\Delta_{tr}}V_\phi^0$ for PTH in the studied systems has been evaluated as follow:

3 $${\Delta_{tr}}V_\varphi^0 = V_\varphi^0\left( {{\text{In}}\;{\text{ternary}}\;{\text{PTH}}/{\text{DESs}}/{\text{ethanol}}\;{\text{solution}}} \right) - V_\varphi^0\left( {{\text{in}}\;{\text{binary}}\;{\text{PTH}}/{\text{ethanol}}\;{\text{solution}}} \right)$$

The partial molar transfer volumes ${\Delta_{tr}}V_\phi^0$ are listed in Table 2. Based on the developed model by Friedman and Krishnan^16,17^, the hydration cospheres overlap in the polar-nonpolar and nonpolar—nonpolar groups decreases the volume while the hydration cospheres overlap between polar groups or two ionic groups enhances volume. The obtained values for the systems studied in this work are negative and decrease with increasing in DESs molalities, which explains the superiority of nonpolar–nonpolar and polar—nonpolar interactions over the rest.

The polynomial equation was applied for the temperature dependence $V_\varphi^0$ values as follow^18^:

4 $$V_\varphi^0 = A + BT + C{T^2}$$

where *A*, *B* and *C* are the parameters of the Eq. (4), which were given in Table 3. The apparent molar isobaric expansion $E_\varphi^0$ was calculated using the derivative relative to the temperature of Eq. (4)^19^:

5 $$E_\varphi^0 = {\left( {\frac{\partial V_\varphi^0}{{\partial T}}} \right)_p} = B + 2CT$$

**Table 3:** The parameters *A*, *B*, *C* and correlation coefficient for the temperature dependence of the $V_\varphi^0$ values.

Systems	Parameters			
	*A*	*B*	10^2^ *C*	*R*^2^ ($V_\varphi^0$)^a^
**PTH in ethanol**	271.93	− 0.36	0.04	0.999
PTH in ternary ethanol solution of ChCl:Gly (0.5 mol kg^-1^)	354.51	− 0.88	0.12	0.996
PTH in ternary ethanol solution of ChCl:Gly (1 mol kg^-1^)	370.17	− 0.99	0.13	0.998
PTH in ternary ethanol solution of ChCl:Gly (1.5 mol kg^-1^)	385.93	− 1.12	0.15	0.993
PTH in ternary ethanol solution of ChCl:EG (0.5 mol kg^-1^)	367.43	− 0.96	0.13	0.987
PTH in ternary ethanol solution of ChCl:EG (1 mol kg^-1^)	386.92	− 1.10	0.15	0.999
PTH in ternary ethanol solution of ChCl:EG (1.5 mol kg^-1^)	406.63	− 1.25	0.17	0.998

Standard uncertainty (*u*) for DESs composition was estimated to be less than 5·10^–2^ mol ratio.

^a^ Correlation coefficient for $V_\varphi^0$ values.

The obtained $E_\varphi^0$ values are reported in Table 4. The structure breaking or making behaviors of the various solutes can be interpreted with the values of $E_\varphi^0$ that directly related to interactions^20^. The all binary PTH/ethanol and ternary PTH/ethanol/DESs have negative $E_\varphi^0$ values. The obtained values for the PTH in the mentioned systems have been increased with rising temperatures.

**Table 4:** The apparent molar isobaric expansions ($$E_{p,\varphi }^0$$) and Hepler’s constants $${\left( {{\raise0.7ex\hbox{${{\partial^2}V_\varphi^0}$} \!\mathord{\left/ {\vphantom {{{\partial^2}V_\varphi^0} {\partial {T^2}}}}\right.\kern-\nulldelimiterspace}\!\lower0.7ex\hbox{${\partial {T^2}}$}}} \right)_p}$$ for binary PTH/ethanol and ternary PTH/ethanol/DESs solutions at *T* = (288.15 to 318.15) K and at ambient pressure (*P* = 871 hPa).

Systems	10^6^$$E_{p,\varphi }^0$$ (m^3^.mol^-1^.K^-1^)				10^2^$${\left( {{\raise0.7ex\hbox{${{\partial^2}V_\varphi^0}$} \!\mathord{\left/ {\vphantom {{{\partial^2}V_\varphi^0} {\partial {T^2}}}}\right.\kern-\nulldelimiterspace}\!\lower0.7ex\hbox{${\partial {T^2}}$}}} \right)_p}$$
	288.15 K	298.15 K	308.15 K	318.15 K	(m^6^.mol^-2^.K^-2^)
PTH in ethanol	− 0.128	− 0.120	− 0.112	− 0.104	0.08
PTH in ternary ethanol solution of ChCl:Gly (0.5 mol kg^-1^)	− 0.2006	− 0.1771	− 0.1536	− 0.1301	0.24
PTH in ternary ethanol solution of ChCl:Gly (1 mol kg^-1^)	− 0.2155	− 0.1885	− 0.1615	− 0.1345	0.27
PTH in ternary ethanol solution of ChCl:Gly (1.5 mol kg^-1^)	− 0.2364	− 0.2059	− 0.1755	− 0.1450	0.30
PTH in ternary ethanol solution of ChCl:EG (0.5 mol kg^-1^)	− 0.2206	− 0.1951	− 0.1695	− 0.1440	0.26
PTH in ternary ethanol solution of ChCl:EG (1 mol kg^-1^)	− 0.2378	− 0.2078	− 0.1778	− 0.1478	0.30
PTH in ternary ethanol solution of ChCl:EG (1.5 mol kg^-1^)	− 0.2553	− 0.2208	− 0.1862	− 0.1517	0.35

Standard uncertainty (*u*) for DESs composition was estimated to be less than 5·10^–2^ mol ratio.

The second derivative of $V_\varphi^0$ relative to temperature is an important quantity to explain the structure breaking or making properties that developed by Hepler as follow^21^:

6 $${\left( {\frac{\partial E_\varphi^0}{{\partial T}}} \right)_p} = {\left( {\frac{{{\partial^2}V_\varphi^0}}{{\partial {T^2}}}} \right)_p} = 2C$$

Table 4 reports the obtained values of $${\left( {{\raise0.7ex\hbox{${{\partial^2}V_\varphi^0}$} \!\mathord{\left/ {\vphantom {{{\partial^2}V_\varphi^0} {\partial {T^2}}}}\right.\kern-\nulldelimiterspace}\!\lower0.7ex\hbox{${\partial {T^2}}$}}} \right)_p}$$ for studied systems. The values of this constant for the all systems are positive that indicates the performance of PTH is as structure making in the presence of ethanol and DESs. The trend for PTH in the presence of DESs is as follows; ChCl:EG ˃ ChCl:Gly.

**Figure 4:**
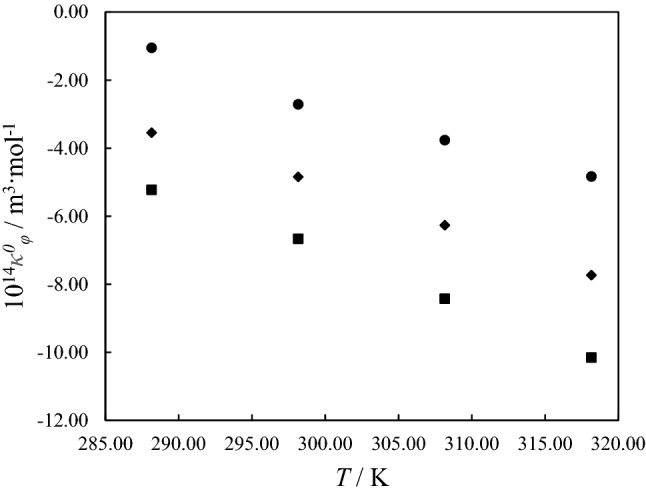
The comparison of the partial molar isentropic compressibility *κ*^*0*^_*φ*_, of PTH in binary PTH/ethanol solutions and ternary PTH/DESs/ethanol solutions with DESs molality = 1 mol/kg at different temperatures: (filled black circle), binary PTH/ethanol solution; (filled black square), ternary PTH/ChCl:EG/ethanol; (filled black diamond), ternary PTH/ChCl:Gly/ethanol.

The experimental density and speed of sound data were used to calculate the isentropic compressibility, *κ*_*s*_ (Pa^-1^). This quantity is due to the resistance of the fluid to changes in pressure and consequently to changes in density and volume. Laplace-Newton’s equation was applied to compute *κ*_*s*_ as follow^22^:

7 $${\kappa_s} = \frac{1}{{\rho {u^2}}}$$

where, the speed of sound is indicated by *u*. The partial molar isentropic compressibilities *κ*_*φ*_, for binary PTH/ethanol and ternary PTH/ethanol/DESs solutions, are calculated as follow^23^:

8 $${\kappa_\varphi } = \frac{{({\kappa_s}{\rho_0} - {\kappa_{s0}}\rho )}}{{m\rho {\rho_0}}} + \frac{{{\kappa_s}M}}{\rho }$$

where, *κ*_*s0*_ is the isentropic compressibility of solvent. The calculated values of *κ*_*φ*_ were reported in Table 5. According to the results in Table 5, it can be seen that the *κ*_*φ*_ values ​​decreased with increasing PTH molalities and also with increasing temperature. The interactions for PTH and solvent can also be explained using these values. Finally, the *κ*_*φ*_ values were correlated using the Redlich-Meyer equation as follow^24^.

9 $${\kappa_\varphi } = \kappa_\varphi^0 + {B_\kappa }m$$

where, *κ*^*0*^_*φ*_ and *B*_*κ*_ are the partial isentropic compressibility and equation parameter, respectively. The obtained parameters are given in Table 6. Figure 4, shows the values of *κ*^*0*^_*φ*_ versus the temperature. This quantity, like the $V_\varphi^0$ expresses PTH-solvent interactions. The $\kappa_\varphi^0$ values are decreased with increasing temperature in the all studied systems.

**Table 5:** Experimental speed of sounds *u* data and partial molar isentropic compressibility,${\kappa_\phi }$ values for PTH molalities *m*_PTH_ (mole of PTH per 1 kg of ethanol for binary system and mole of PTH per 1 kg of DESs/ethanol solutions for ternary system) in binary PTH/ethanol and ternary PTH/DESs (ChCl:Gly and ChCl:EG)/ethanol solutions at *T* = (288.15 to 318.15) K and ambient pressure (*P* = 871 hPa).

*m /* mol kg^-1^	*u* / m s^-1^				10^14^ *κ*_*φ*_ / m^3^ mol^-1^ Pa^-1^			
*T /* K	288.15	298.15	308.15	318.15	288.15	298.15	308.15	318.15
**PTH in Ethanol**								
0.0206	1178.32	1144.13	1110.23	1077.00	− 2.58	− 4.01	− 5.11	− 6.37
0.0307	1178.93	1144.73	1110.83	1077.55	− 3.30	− 4.68	− 5.94	− 6.95
0.0411	1179.60	1145.40	1111.50	1078.17	− 3.93	− 5.36	− 6.76	− 7.67
0.0525	1180.54	1146.27	1112.28	1078.92	− 5.11	− 6.37	− 7.58	− 8.61
0.0606	1181.12	1146.79	1112.88	1079.45	− 5.49	− 6.63	− 8.22	− 9.15
0.0697	1181.90	1147.49	1113.50	1080.11	− 6.12	− 7.14	− 8.50	− 9.79
**PTH in ternary ethanol solution of ChCl:Gly (0.5 mol kg**^**-1**^**)**								
0.0000								
0.0201	1196.63	1163.50	1130.27	1096.99	− 3.54	− 4.50	− 5.59	− 6.12
0.0293	1197.19	1164.03	1130.75	1097.46	− 3.96	− 4.87	− 5.76	− 6.61
0.0391	1197.90	1164.71	1131.40	1098.03	− 4.84	− 5.87	− 6.85	− 7.59
0.0516	1198.89	1165.61	1132.23	1098.76	− 5.89	− 6.81	− 7.89	− 8.49
0.0585	1199.43	1166.08	1132.70	1099.20	− 6.39	− 7.20	− 8.29	− 9.10
0.0697	1200.35	1166.97	1133.44	1099.90	− 7.15	− 8.16	− 8.97	− 9.85
**PTH in ternary ethanol solution of ChCl:Gly (1 mol kg**^**-1**^**)**								
0.0000								
0.0202	1213.28	1180.84	1147.98	1115.37	− 4.40	− 5.80	− 7.27	− 8.92
0.0306	1214.00	1181.60	1148.70	1116.05	− 4.83	− 6.53	− 7.84	− 9.26
0.0423	1214.82	1182.41	1149.50	1116.90	− 5.33	− 6.91	− 8.31	− 10.10
0.0519	1215.53	1183.14	1150.18	1117.55	− 5.79	− 7.44	− 8.76	− 10.50
0.0596	1216.05	1183.71	1150.75	1118.14	− 5.97	− 7.80	− 9.19	− 11.00
0.0697	1216.87	1184.53	1151.57	1118.90	− 6.53	− 8.41	− 9.80	− 11.53
**PTH in ternary ethanol solution of ChCl:Gly (1.5 mol kg**^**-1**^**)**								
0.0000								
0.0213	1228.92	1196.85	1164.46	1132.73	− 5.13	− 6.80	− 8.44	− 10.16
0.0302	1229.58	1197.54	1165.15	1133.42	− 5.35	− 7.12	− 8.77	− 10.56
0.0387	1230.21	1198.25	1165.82	1134.09	− 5.55	− 7.56	− 9.03	− 10.80
0.0494	1231.05	1199.15	1166.71	1134.93	− 5.97	− 8.03	− 9.54	− 11.25
0.0614	1232.05	1200.16	1167.73	1135.93	− 6.42	− 8.43	− 10.01	− 11.71
0.0700	1232.69	1200.94	1168.45	1136.59	− 6.62	− 8.82	− 10.26	− 11.83
**PTH in ternary ethanol solution of ChCl:EG (0.5 mol kg**^**-1**^**)**								
0.0000								
0.0202	1193.79	1159.65	1126.97	1093.46	− 4.89	− 5.27	− 6.12	− 6.61
0.0306	1194.49	1160.30	1127.55	1093.99	− 5.48	− 5.97	− 6.63	− 7.17
0.0424	1195.37	1161.06	1128.26	1094.64	− 6.40	− 6.68	− 7.44	− 7.99
0.0520	1196.14	1161.74	1128.85	1095.16	− 7.04	− 7.33	− 7.96	− 8.55
0.0609	1196.84	1162.43	1129.43	1095.67	− 7.56	− 8.10	− 8.51	− 9.16
0.0691	1197.58	1163.01	1130.00	1096.21	− 8.32	− 8.57	− 9.10	− 9.84
**PTH in ternary ethanol solution of ChCl:EG (1 mol kg**^**-1**^**)**								
0.0202	1207.46	1174.89	1142.08	1109.49	− 5.72	− 7.14	− 8.92	− 10.65
0.0306	1208.18	1175.62	1142.80	1110.23	− 6.06	− 7.50	− 9.23	− 11.15
0.0423	1208.98	1176.42	1143.60	1111.03	− 6.33	− 7.79	− 9.44	− 11.42
0.0519	1209.67	1177.11	1144.29	1111.68	− 6.66	− 8.11	− 9.84	− 11.70
0.0596	1210.18	1177.62	1144.81	1112.23	− 6.77	− 8.24	− 10.01	− 11.96
0.0697	1210.86	1178.3	1145.48	1112.91	− 7.04	− 8.45	− 10.14	− 12.15
**PTH in ternary ethanol solution of ChCl:EG (1.5 mol kg**^**-1**^**)**								
0.0000								
0.0198	1220.60	1188.97	1157.02	1123.70	− 6.71	− 8.27	− 10.01	− 12.08
0.0313	1221.53	1189.9	1157.95	1124.63	− 7.25	− 8.68	− 10.54	− 12.59
0.0436	1222.58	1190.95	1159	1125.68	− 7.77	− 9.28	− 11.07	− 13.23
0.0539	1223.44	1191.81	1159.86	1126.54	− 8.01	− 9.56	− 11.36	− 13.57
0.0599	1223.92	1192.29	1160.34	1127.02	− 8.12	− 9.64	− 11.43	− 13.63
0.0683	1224.71	1193.08	1161.13	1127.81	− 8.50	− 10.01	− 11.87	− 14.11

Standard uncertainties (*u*) for each variable are *u* (*T)* = 0.001 K; *u* (*m*) = 0.0005 mol kg^-1^; *u* (*p*) = 10 hPa.

The combined standard uncertainty for the average of n speed of sound measurements *u* (*u*) = 1 m s^-1^.

Standard uncertainty (*u*) for DESs composition was estimated to be less than 5·10^–2^ mol ratio.

^a^*m* is the molality of PTH, mole of PTH per 1 kg of solvents.

**Table 6:** The obtained partial molar isentropic compressibility *κ*^*0*^_*φ*_, experimental parameters *B*_*k*_, and ∆_tr_*κ*^*0*^_*φ*_ along standard deviations *σ* (*κ*_*φ*_) for binary PTH/ethanol and ternary PTH/ethanol/DESs solutions at *T* = (288.15 to 318.15) K and at ambient pressure (*P* = 871 hPa).

*T* / K	10^14^ *κ*^*0*^_*φ*_ / m^3^ mol^-1^ Pa^-1^	10^14^ *B*_*k*_ / kg m^3^ mol^-2^ Pa^-1^	10^14^ ∆_tr_*κ*^*0*^_*φ*_ / m^3^ mol^-1^ Pa^-1^	*σ* (*κ*_*φ*_)
**PTH in ethanol**				
288.15	− 1.05	− 73.63	–	0.12
298.15	− 2.71	− 65.09	–	0.13
308.15	− 3.76	− 71.08	–	0.16
318.15	− 4.83	− 71.05	–	0.07
**PTH in ternary ethanol solution of ChCl:Gly (0.5 mol kg**^**-1**^**)**				
288.15	− 1.90	− 75.83	− 0.85	0.11
298.15	− 2.86	− 75.57	− 0.15	0.13
308.15	− 3.94	− 73.47	− 0.18	0.21
318.15	− 4.49	− 77.56	0.34	0.10
**PTH in ternary ethanol solution of ChCl:Gly (1 mol kg**^**-1**^**)**				
288.15	− 3.54	− 42.28	− 2.49	0.06
298.15	− 4.84	− 50.47	− 2.13	0.09
308.15	− 6.26	− 49.74	− 2.50	0.07
318.15	− 7.73	− 54.31	− 2.90	0.09
P**TH in ternary ethanol solution of ChCl:Gly (1.5 mol kg**^**-1**^**)**				
288.15	− 4.40	− 32.00	− 3.35	0.06
298.15	− 5.92	− 41.48	− 3.21	0.05
308.15	− 7.60	− 38.55	− 3.84	0.05
318.15	− 9.45	− 35.34	− 4.62	0.08
**PTH in ternary ethanol solution of ChCl:EG (0.5 mol kg**^**-1**^**)**				
288.15	− 3.42	− 69.69	− 2.37	0.08
298.15	− 3.87	− 68.05	− 1.16	0.07
308.15	− 4.83	− 60.95	− 1.07	0.06
318.15	− 5.22	− 65.56	− 0.39	0.08
**PTH in ternary ethanol solution of ChCl:EG (1 mol kg**^**-1**^**)**				
288.15	− 5.22	− 26.42	− 4.17	0.04
298.15	− 6.66	− 26.60	− 3.95	0.06
308.15	− 8.42	− 25.73	− 4.66	0.07
318.15	− 10.15	− 29.64	− 5.32	0.08
**PTH in ternary ethanol solution of ChCl:EG (1.5 mol kg**^**-1**^**)**				
288.15	− 6.11	− 35.13	− 5.06	0.10
298.15	− 7.61	− 35.35	− 4.90	0.08
308.15	− 9.37	− 36.41	− 5.61	0.10
318.15	− 11.33	− 40.50	− 6.50	0.10

Standard uncertainty (*u*) for DESs composition was estimated to be less than 5·10^–2^ mol ratio.

The partial molar transfer isentropic compressibility ${\Delta_{tr}}\kappa_\phi^0$ for PTH in the systems is obtained as follow:

10 $${\Delta_{tr}}\kappa_\phi^0 = \kappa_\phi^0\;\left( {{\text{In}}\;{\text{ternary}}\;{\text{PTH}}/{\text{DESs}}/{\text{ethanol}}\;{\text{solution}}} \right) - \kappa_\phi^0\left( {{\text{in}}\;{\text{binary}}\;{\text{PTH}}/{\text{ethanol}}\;{\text{solution}}} \right)$$

These ${\Delta_{tr}}\kappa_\phi^0$ values are listed in Table 8.

The standard deviation (*σ*) is applied to check the adaptability of the experimental values by the obtained values with the Redlich-Meyer equation using the subsequent equation:

11 $$\sigma (X) = \sqrt {\frac{{\sum\limits_{i = 0}^{last(m)} {{{(X_i^{\exp } - X_i^{cal})}^2}} }}{N - n}}$$

where $X_i^{\exp }$, $X_i^{cal}$, *n*, and *N* are introduced as the experimental and calculated values of ${V_\varphi }$ and ${\kappa_\varphi }$ values*,* the number of parameters and experimental points, respectively. The values of *σ* for the all studied systems are given in Tables 2 and 6.

### Hansen solubility parameters results

Hansen solubility parameters are one of the most important methods for investigation of solute interaction in the presence of solvent. With these parameters, the appropriate solvent can be selected. Hildebrand first introduced solubility parameters that "similar solves similar"^25^. This parameter is modified by Hansen^26^ and is used as the Hildebrand-Hansen parameter. Solubility parameters are determined experimentally or by calculations as follow:

12 $${\delta^2} = \frac{{{E_{coh}}}}{{V_m}} = \frac{{\Delta {H_{vap}} - RT}}{{V_m}}$$

where Δ*H*_vap_, *V*_m_, and *E*_coh_ are the evaporation enthalpy, the molar volume and the intermolecular forces (adhesion energy), respectively. Also, *R* and *T* are the general constant of the gases and the temperature.

The introduced solubility parameter is expressed as follow; failure of hydrogen bonds between molecules (*δ*_h_), adjacent intermolecular forces (bipolar interactions) (*δ*_p_), and adhesion energy density, from the sum of energies required to overcome scattering forces (*δ*_d_):

13 $$\delta_t^2 = \delta_d^2 + \delta_p^2 + \delta_h^2$$

The mutual solubility between solute *i* and solvent *j* is calculated as follow:

14 $$\Delta {\delta_{ij}} = \sqrt {4{{(\delta_d^i - \delta_d^j)}^2} + {{(\delta_p^i - \delta_p^j)}^2} + {{(\delta_h^i - \delta_h^j)}^2}}$$

to determine *δ*_h_, *δ*_p_, and *δ*_d_, methods based on structural contributions of functional groups are used. Thus, *δ*_d_ is estimated from the following relation:

15 $${\delta_d} = \frac{{\sum {F_d} }}{{V_m}}$$

where *F*_d_ is the constant dispersion component of molar adsorption. The interactions of polar groups are also expressed by using the following equation:

16 $${\delta_p} = \frac{{\sqrt {{{\sum {F_p} }^2}} }}{{V_m}}$$

where, *F*_p_ is the constant polar component of molar adsorption. *δ*_h_ can also be determined as follow:

17 $${\delta_h} = \frac{{\sqrt {\sum {E_h} } }}{{V_m}}$$

where *E*_h_ is the hydrogen bond adhesion energy per structural group. Using the literature^27^, we can estimate the solubility parameters for DESs (ChCl:EG and ChCl:Gly), PTH and ethanol.

In this study, the parameters *δ*_d_, *δ*_p_ and *δ*_h_ were estimated from sources and some were obtained using the Krevelen and Hoftyzer method^28,29^ for PTH drug, DESs and ethanol, which are collected in the Table 7. Differences between drug solubility parameter and solvents (ethanol and ethanol/DESs) are calculated from Eq. (14) and are reported in the Table 8. As can be seen from the results in Table 8, $\Delta \delta$ values indicating a strong interaction between PTH and solvent (ethanol/DES (ChCl:EG)) relative to others systems.

**Table 7:** The calculated Hildebrand-Hansen solubility parameters for the materials used by Hoftyzer and Van Krevelen method^29^.

Systems	*δ*_d_	*δ*_p_	*δ*_h_	*δ*t
PTH	23.93	7.39	8.133	26.332
Ethanol	15.80	8.80	19.40	26.522
Ethanol/DES (ChCl:Gly)	17.31	5.05	22.07	28.496
Ethanol/DES (ChCl:EG)	16.47	4.86	19.74	26.169

**Table 8:** The calculated ∆δ for PTH drug and solvents (ethanol and ethanol/DESs).

Systems solute	ethanol	ethanol/ChCl:Gly	ethanol/ChCl:EG
Phenytoin (PTH)	19.832	19.363	19.066

## Experimental

### Chemicals

Choline chloride (GR, 0.998), ethylene glycol (GR, 0.999), glycerol (GR, 0.998), and ethanol (GR, 0.998) were purchused from Merck Co. Phenytoin (PTH) in mass fraction (> 0.99) is purchased from Daana Pharm. Co. (Tabriz, Iran). All chemicals used are reagent grade without further purification. Table 1 summarized the information of the chemicals applied in this work. It should be mentioned that the purity of the all chemicals is provided by the suppliers (Table 9).

**Table 9:** A summary of the used chemicals.

Chemical name	Abbreviation	Supplier	CAS No	Mass fraction (purity)	Structure
Phenytoin	PTH	Daana Pharm. Co. Iran	57–41-0	> 0.99	
Choline Chloride	ChCl	Merck	67–48-1	> 0.99	
Ethylene Glycol	EG	Merck	107–21-1	> 0.99	
Glycerol	Gly	Merck	56–81-5	> 0.99	
Ethanol	−	Merck	64–17-5	> 0.99	

The suppliers were provided the purities of the used components.

The purified compounds of EG or Gly as HBDs and ChCl as HBA were mixed with the molar ratio 1:2 in the water bath at temperature about 333 K for 4 h until a colorless and homogeneous liquid formed^11^. For the prepared DESs composition, the uncertainty of less than 5·10^–2^ mol was estimated. Using the Karl − Fisher titration technique (method TitroLine KF), the water content was measured for the prepared DESs. Eventually, a vacuum pump was used to remove moisture and excess impurities of the DESs. Some of the properties of DESs (ChCl:Gly and ChCl:EG) are listed in Table 10.

**Table 10:** Some of the physical properties of DESs (binary mixtures) used in the work at 298.15 K and pressure (*p* = 871 hPa).

	Molar ratio	Melting Point (K)	Water content (w%)	Molar mass (g mol^-1^)^a^	*T* / K	*ρ* / g cm^-3^ (Exp)	*ρ* / g cm^-3^ (Lit)	*u* / m s^-1^ (Exp)	*u* / m s^-1^ (Lit)
ChCl:EG	1:2	207.15^30^	< 0.01%	87.921	298.15	1.115551	1.115616^31^	1909.20	1909.65^31^
							1.138^32^		1911.04^30^
									1905.1^33^
					303.15	1.112750	1.112715^31^	1897.43	1897.48^31^
									1894.0^33^
								1885.63	1914^32^
					308.15	1.109927	1.109927^31^		1886.08^31^
									1882.8^33^
					313.15	1.107151	1.1084^34^	1873.86	1882^32^
							1.1057^35^		1871.8^33^
					318.15	1.104361	1.10529^36^	1861.58	1860.7^33^
ChCl:Gly	1:2	233.15^30^	0.06%	107.937	298.15	1.186358	1.1920^37^	2012.42	2012.59^30^
							1.19085^38^		
							1.181^32^		
							1.19575^39^		2001.29^39^
					303.15	1.183556	1.1895^37^	2001.05	2080^32^
							1.18807^38^		
							1.19290^39^		1990.23^39^
					308.15	1.180849	1.1867^37^	1989.90	
							1.18528^38^		
							1.19015^39^		1979.24^39^
					313.15	1.178128	1.1838^37^	1978.98	1976^32^
							1.18249^38^		
							1.18740^39^		1968.30^39^
					318.15	1.175437	1.1814^37^	1967.90	
							1.17970^38^		
							1.18465^39^		1957.38^39^

Standard uncertainties (*u*) for each variable are *u* (*T)* = 0.001 K; *u* (*p*) = 10 hPa.

The combined standard uncertainty for the average of n density measurements *u* (*ρ)* = 0.015 kg m^-3^ and speed of sound *u* (*u*) = 1 m s^-1^.

Standard uncertainty (*u*) for DESs composition was estimated to be less than 5·10^–2^ mol ratio.

^a^ Molar mass of DESs = *x*_1_ M_1_ + *x*_2_ M_2_.

*x*_1_ and M_1_; mole fraction and molar mass of ChCl.

*x*_2_ and M_2_; mole fraction and molar mass of HBD.

The melting point is expressed for the solidus (formation of the first liquid) or liquids (disappearance of last crystals).

The density and speed of sound were measured for the liquid state of the prepared DESs.

### Apparatus and procedure

All solutions were prepared by filling tight glass vials, which are containing different amounts of the PTH in the water and ternary DESs solutions. In this regard, an analytical balance with precision 10^–4^ g (AW 220, GR220, Shimadzu, Japan) was used.

The molality of PTH was introduced as follows; mole of PTH per kg of solvent (binary in ethanol and ternary in DESs/ethanol solutions). For all of the prepared solutions, the uncertainty was estimated to be less than 5·10^–4^ mol·kg^-1^.

Density and speed of sound measuring device of Anton Paar Co. (with model DSA 5000, Austria) at the frequency (approximately 3 MHz) was utilized for all the binary (PTH/ethanol) and ternary (PTH/DESs/ethanol) solutions. After washing the device with deionized water and ethanol and drying with air, the device was calibrated using degassed and deionized water at the *T* = 293.15 K and atmospheric pressure. A Peltier device embedded inside the apparatus has been utilized to keep the temperature of the samples with an accuracy of 0.001 K. The standard uncertainties for density and speed of sound measurements were estimated to be 0.015 kg m^-3^ and 1 m s^-1^, respectively^20^. The measured data for the DESs used in this work were compared with the data reported in the literature and are given in Table 10. The data are well matched and in an acceptable range. Uncertainties are also given for the data reported in the relevant tables.

## Conclusions

The most important part of drug preparation and production is the investigation of the interactions that occur between the drug and the solvent. In this regard, the volumetric and compressibility properties were applied to describe these interactions. As can be understood from the results of $V_\varphi^0$ and $\kappa_\varphi^0$ values, the interaction between PTH and ethanol molecules has increased with increasing DESs molality and temperature. The results represent stronger interactions for DES (ChCl:EG). The Hepler values for the systems are positive that indicating the performance of PTH is studied as structure making in ethanol and in the presence of DESs solutions. The trend of this behavior for the PTH in presence of DESs as follows: ChCl:EG ˃ ChCl:Gly. The experimental results and the Hansen solubility parameters are very well compatible. Experimental and calculations results indicating a strong interaction between PTH and solvent (ethanol:DES (ChCl:EG *m* = 1.5 mol kg^-1^)) than the other systems.
